# Experience-based human perception of facial expressions in Barbary macaques (*Macaca sylvanus*)

**DOI:** 10.7717/peerj.3413

**Published:** 2017-06-01

**Authors:** Laëtitia Maréchal, Xandria Levy, Kerstin Meints, Bonaventura Majolo

**Affiliations:** School of Psychology, University of Lincoln, Lincoln, Lincolnshire, United Kingdom

**Keywords:** Facial expressions, Primates, Ethnoprimatology, Tourism, Human-macaque interactions, Universal hypothesis

## Abstract

**Background:**

Facial expressions convey key cues of human emotions, and may also be important for interspecies interactions. The universality hypothesis suggests that six basic emotions (anger, disgust, fear, happiness, sadness, and surprise) should be expressed by similar facial expressions in close phylogenetic species such as humans and nonhuman primates. However, some facial expressions have been shown to differ in meaning between humans and nonhuman primates like macaques. This ambiguity in signalling emotion can lead to an increased risk of aggression and injuries for both humans and animals. This raises serious concerns for activities such as wildlife tourism where humans closely interact with wild animals. Understanding what factors (i.e., experience and type of emotion) affect ability to recognise emotional state of nonhuman primates, based on their facial expressions, can enable us to test the validity of the universality hypothesis, as well as reduce the risk of aggression and potential injuries in wildlife tourism.

**Methods:**

The present study investigated whether different levels of experience of Barbary macaques, *Macaca sylvanus*, affect the ability to correctly assess different facial expressions related to aggressive, distressed, friendly or neutral states, using an online questionnaire. Participants’ level of experience was defined as either: (1) naïve: never worked with nonhuman primates and never or rarely encountered live Barbary macaques; (2) exposed: shown pictures of the different Barbary macaques’ facial expressions along with the description and the corresponding emotion prior to undertaking the questionnaire; (3) expert: worked with Barbary macaques for at least two months.

**Results:**

Experience with Barbary macaques was associated with better performance in judging their emotional state. Simple exposure to pictures of macaques’ facial expressions improved the ability of inexperienced participants to better discriminate neutral and distressed faces, and a trend was found for aggressive faces. However, these participants, even when previously exposed to pictures, had difficulties in recognising aggressive, distressed and friendly faces above chance level.

**Discussion:**

These results do not support the universality hypothesis as exposed and naïve participants had difficulties in correctly identifying aggressive, distressed and friendly faces. Exposure to facial expressions improved their correct recognition. In addition, the findings suggest that providing simple exposure to 2D pictures (for example, information signs explaining animals’ facial signalling in zoos or animal parks) is not a sufficient educational tool to reduce tourists’ misinterpretations of macaque emotion. Additional measures, such as keeping a safe distance between tourists and wild animals, as well as reinforcing learning via videos or supervised visits led by expert guides, could reduce such issues and improve both animal welfare and tourist experience.

## Introduction

The ability to interpret the emotional states of others correctly is one of the key aspects of primate social communication, and facial expressions have been shown to be informative in this regard (Hinde & Rowell, 1962; Ekman, 1993; Leopold & Rhodes, 2010). In humans, the ability to categorise emotions based on their facial expressions differs with age and across emotions, with difficulty increasing from happiness, sadness and anger, to fear and disgust, to neutral emotional states (Durand et al., 2007). Some studies have shown that nonhuman primates can discriminate the facial expressions of their conspecifics. For example, chimpanzees were able to match positive or negative valence stimuli to corresponding facial expressions (Parr, 2001). Other studies have shown that Japanese, rhesus and crested macaques were overall able to discriminate conspecific facial expressions by using a matching-to-sample procedure (Kanazawa, 1996; Parr & Heintz, 2009; Micheletta et al., 2015).

Although facial expressions convey an effective means of communication of one’s own emotions to others within a species, they may also be used and generalised to facilitate interspecific interactions. For instance, chimpanzees, dogs, cats and horses have been shown to discriminate facial expressions of positive and negative emotions in humans, which can facilitate the interactions between humans and animals (Buttelmann, Call & Tomasello, 2009; Müller et al., 2015; Galvan & Vonk, 2016; Smith et al., 2016). Six basic emotions (anger, disgust, fear, happiness, sadness, and surprise) have been suggested to be universally recognised across cultures in humans (Ekman, 1992; Ekman, 1993). According to the universality hypothesis (Darwin, 1872; Darwin, Ekman & Prodger, 1998), the facial expression of the six basic emotions is homologous in humans and nonhuman primates, both in terms of their morphological features and their social function (Preuschoft & Van Hooff, 1997; Julle-Daniere et al., 2015; Micheletta et al., 2015). However, some facial expressions may seem morphologically similar in human and nonhuman primates but stem from different emotional states (Preuschoft, 1992; Preuschoft & Van Hooff, 1997; Leopold & Rhodes, 2010). For example, the human smile is linked to an emotional state of happiness, which is expressed in non-aggressive or friendly contexts (Kraut & Johnston, 1979). In a number of nonhuman primate species, such as macaques, animals display a facial expression apparently similar to a human smile called the ‘bared-teeth’. This facial expression is, however, related to a distressed emotional state (including appeasement, fear or anxiety), and it is usually displayed as a submissive behaviour during aggressive or dominant interactions (Preuschoft, 1992; Preuschoft & Van Hooff, 1997). Such differences in the function and context of apparently similar displays of facial expressions suggest that phenotypic similarity of facial expressions in the primate order may be caused by different emotional states and/or be displayed in different contexts.

The Biophilia hypothesis (Wilson, 1984) suggests that humans have an innate emotional attraction towards animals, which is thought to explain the predilection of humans for interspecies interactions. The misinterpretation between positive and negative valence associated with similar facial expressions in humans and nonhuman primates can increase the risks of aggression and of potential injuries for both humans and animals involved in this interspecies interface. This is a particular concern as interspecies interactions such as wildlife tourism are increasing. Wildlife tourism is a growing international industry, where close interactions with wild animals such nonhuman primates often occur. Tourists are observed attempting to be in close proximity to and interact with these animals by touching or feeding them (Orams, 2002; Maréchal et al., 2011; Maréchal et al., 2016a; Maréchal et al., 2016b). However, such close interactions with wild nonhuman primates, such as Barbary macaques, have been shown to increase the risks of aggression and of potential injuries for both tourists and animals (Zhao, 2005; Knight, 2011; Maréchal et al., 2011; Majolo et al., 2013). Tourists may not be able to assess the animal’s emotional state correctly, misinterpret it, and behave towards the animal in inappropriate ways, hence increasing their risk of injury. Indeed, nonhuman primates’ aggression towards tourists is often elicited by human behaviour, such as sudden gestures (McCarthy et al., 2009; Beisner et al., 2015). Therefore, this misunderstanding of an animal’s emotional state could potentially be an important cause of bites tourists receive from monkeys in different primate tourism locations such as the Cape peninsula (South Africa), the Gibraltar rock (UK), and temples in South-East Asia as often reported in popular media (WHO, 2013; Riesland & Wilde, 2015).

Being able to identify the emotional states of animals that people interact with, based on their behaviour or facial expressions, may reduce the risk of injury. Experience with a specific animal species is an important factor that increases the ability to recognise emotional states in that species based on their behaviour (Diesel, Brodbelt & Pfeiffer, 2008; Tami & Gallagher, 2009; Kujala et al., 2012; Mirkó, Dóka & Miklósi, 2013). Experience also appears to have a small effect on the ability to recognise animal’ emotions based on their facial expressions (Bloom & Friedman, 2013; Schirmer, Seow & Penney, 2013; Kujala et al., 2017). For example, professional dog trainers have higher performance in recognising dogs’ emotions based on their behaviour than dog owners with no proper behavioural training, and lay people (e.g., Tami & Gallagher, 2009; Wan, Bolger & Champagne, 2012).

Although level of experience might be a key factor for humans to assess animal emotion, some nonhuman animal emotional states also seem to be more difficult to recognise than others (Wan, Bolger & Champagne, 2012; Bloom & Friedman, 2013). For instance, dogs’ happiness was easily recognised based on their facial expressions by experienced and inexperienced people compared to other emotional states such as sad, neutral or fear faces (Bloom & Friedman, 2013). Indeed, children often misinterpret an aggressive display of teeth in dogs as a smile and misjudge their emotional expression as “happy” (Meints, Racca & Hickey, 2011).

In the present study the term “experience” refers to the knowledge acquired on Barbary macaques’ behaviour and facial expressions either by a brief exposure to pictures (low experience) or by working with macaques over an extended period of time (high experience). To our knowledge, the ability to categorise macaque emotion based on their facial expressions has not been tested as a function of experience and for different emotional states. It is also unknown whether people might increase their performance after simple exposure to 2D pictures of different Barbary macaques’ facial signalling. By investigating interspecies ability to assess emotional states based on facial expressions, as well as experience based recognition, our aims were to: (1) test the validity of the universality hypothesis using phylogenetically closely related species, human and nonhuman primates; (2) apply our findings to judge the potential risk of injuries at primate tourism sites due to tourists being unable to successfully recognise the facial expressions of the animals.

To assess people’s ability to interpret nonhuman primates’ emotional states correctly based on their facial expressions, we conducted an online questionnaire (see Supplemental Information 2) where participants with different levels of experience with Barbary macaques were shown pictures of different macaques’ facial signalling in order to test the following hypotheses: (1) Experience with Barbary macaques is related to high performance in assessing macaque emotion; (2) Aggressive and distressed faces can be mistaken with neutral or friendly faces, particularly for inexperienced people. The latter would be particularly problematic as misinterpreting a threatening display from Barbary macaques as a positive stimulus may lead to physical aggression and injury for tourists.

## Method

This study was approved by the University of Lincoln’s School of Psychology Research Ethics Committee.

### Barbary macaque tourism as a case study for interspecies interactions

Barbary macaques are currently present in Morocco, Algeria and Gibraltar, where they are a popular tourist attraction (Perez & Bensusan, 2005; Majolo et al., 2013). Interactions between Barbary macaques and tourists/locals are a major concern in Gibraltar (Perez & Bensusan, 2005) and are a growing issue in many tourist destinations in Morocco (Maréchal et al., 2011; Maréchal et al., 2016a; Maréchal et al., 2016b). The species was classified in 2008 as Endangered by the IUCN (Butynski et al., 2008), and primate tourism is thought to have the potential to be beneficial for the conservation of the Barbary macaque by increasing public awareness, providing protection for the species and their habitats, and contributing to the local and/or national economy (Russon & Wallis, 2014). However, to be successful, primate tourism must ensure the safety of the tourists and minimise the costs to animal welfare. Therefore, it is important to explore people’s ability to assess Barbary macaques’ emotional states correctly based on their facial expressions. Such research is crucial to help inform policy-making and guide the regulation of primate tourism as well as, inform management decisions in tourist destinations, for example, to assess whether and how close tourists might approach macaques to reduce the risk of injury.

### Participants

In order to test our hypotheses, we collected data on how three groups of participants (*N* = 124) rated the emotional state of a series of macaque facial expressions: (1) naïve participants (*N* = 34, 10 men and 24 women); (2) exposed participants (*N* = 46, 19 men and 27 women); (3) experts (*N* = 44, 12 men and 32 women). Participants were assigned to one of these three groups on the basis of the following criteria: (1) naïve participants were those who had never worked with nonhuman primates, but might have encountered them on rare occasions when visiting zoos, animal parks or in their natural habitat; (2) Exposed participants were naïve participants who we exposed to different Barbary macaques’ facial expressions during the experiment (see section on exposure procedure below); (3) Experts were participants with knowledge about macaque behaviour acquired while working with macaques over a prolonged period of time (at least two months) either in the wild or in captivity (e.g., researchers). Participants were grouped into four categories (18–25, 26–40, 41–60 and +61 years old), with the majority of participants being in the 26–40 years old category (naïve and expert: 72.7%; exposed: 46.6%). All age categories were collapsed together for the purpose of analysis. From a total of 285 participants, we excluded 161 participants from the analyses who: (1) did not fully complete the questionnaire (*N* = 102), (2) had less than two month experience with Barbary macaques (*N* = 11), (3) did not work with Barbary macaques but worked with other nonhuman primate species, as they could not be classified as either naïve or expert participants (*N* = 48). Naïve and exposed participants were recruited via social media, and were randomly assigned to each condition without demographic selection. Expert participants were recruited via social media and professional networks. The selection of participation inclusion was done after data collection, which explains the high rate of participants excluded from the analyses presented below.

### Stimuli

**Figure 1:**
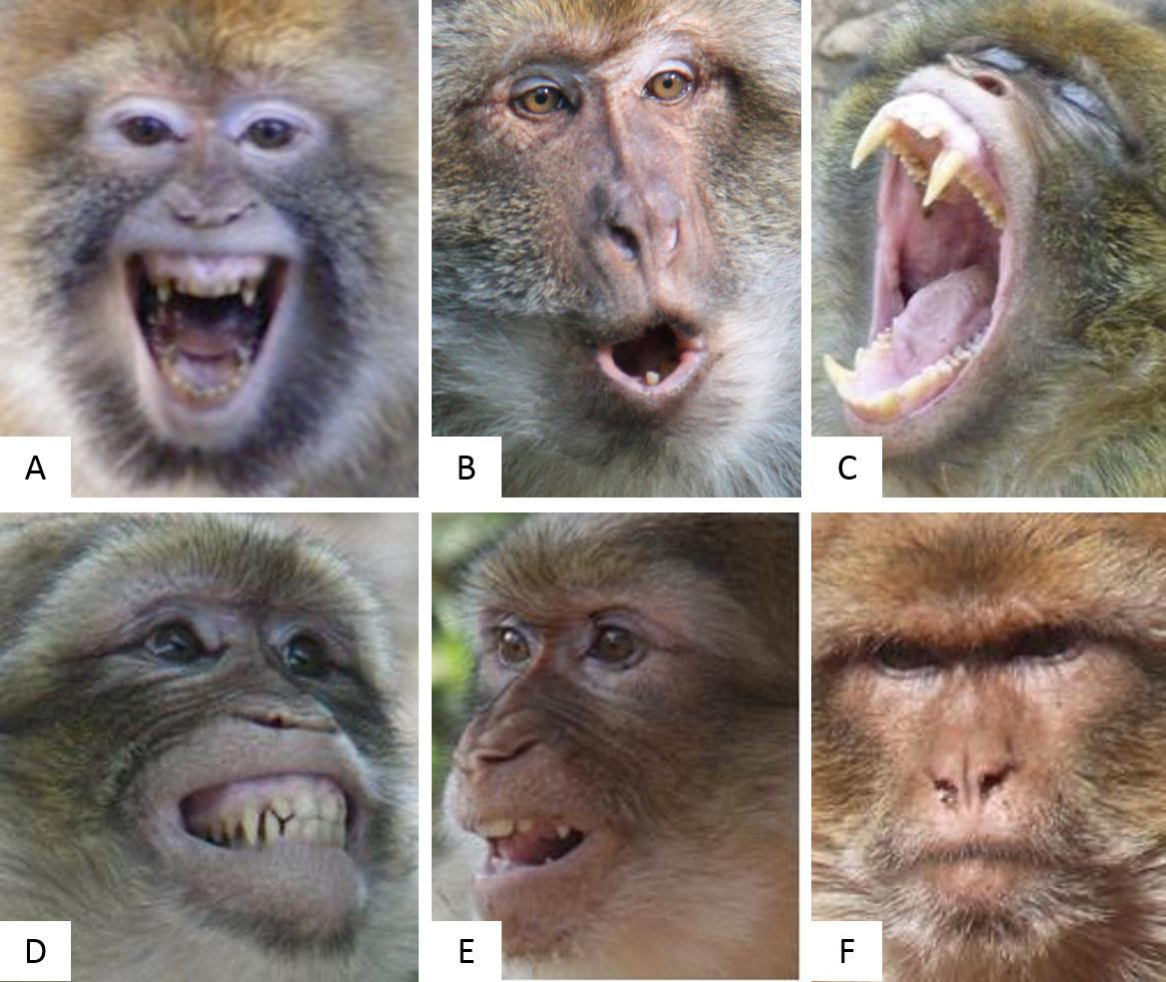
The six pictures presenting the six Barbary macaques’ facial expressions related to the four emotional stated tested. (A) and (B) ‘aggressive’ or ‘threat’ face; (C) and (D) ‘distressed’ or ‘submissive’ face; (E) ‘friendly’ or ‘affiliative’ face; (F) ‘neutral’ face. The description of each facial expression is detailed in the text. Photo credit: Laëtitia Maréchal (A, C), Julia Fischer (B, D, E), and Andrew Forsyth (F).

Each picture showed a face of an adult male or female Barbary macaque. A total of 22 pictures were used in the study, including 16 pictures used for the questionnaire (i.e., four pictures for each emotional state) and presented to every participant, and a series of six additional pictures that were presented only to exposed participants (see exposure procedure, Fig. 1). The pictures used in the present study were high resolution 2D pictures, and came from different sources who all gave permission to use their pictures. All pictures were independently labelled by two experts on Barbary macaques (LM and BM). Of the 22 pictures used, the facial expression and underlying emotion depicted in 16 pictures was independently confirmed by two experts on Barbary macaques (LM and Prof. Julia Fischer). Also, in order to have four pictures in each category, we added six pictures taken by professional photographers (Roger Eritja and Andrew Forsyth), for which the context of the picture was not known. These pictures were selected only if they contained clearly recognisable emotions, i.e., aggressive and neutral, and presented no ambiguity in their interpretation by the experts (LM and BM).

We selected six facial expressions associated with four common emotional states described below. The detailed description of the affiliative, submissive and threat faces can be found in Hesler & Fischer (2008).

(A–B) ‘Aggressive’ or ‘Threat’ face: In the first picture (A), the eyebrows are raised, the animal stares intently and the mouth is open showing the teeth. In the second picture (B), the eyebrows are raised, the animal stares intently and the lips are protruded to form a round mouth;

(C–D) ‘Distressed’ or ‘Submissive’ face: In the first picture (C), the mouth is widely open, and the animal is yawning. Yawing can be related to distress and anxiety in primates (Maestripieri et al., 1992). In the second picture (D), the corners of the lips are fully retracted and the upper and lower teeth are shown;

(E) ‘Friendly’ or ‘Affiliative’ face: In picture (E), the mouth is half open and the lips slightly protruded. This expression involves a chewing movement and clicking or smacking of the tongue and lips;

(F) ‘Neutral’ face: In picture (F), the mouth is closed and the overall face is relaxed.

Each picture of the Barbary macaques’ facial expressions was chosen to show both eyes, the nose and the mouth. This was done so that participants had access to the same amount of information on each image to be able to determine the macaque’s emotional state. Head orientation (horizontally or vertically) could not vary by more than 90° (e.g., Fig. 1), and all pictures were taken in natural daylight and with natural background slightly visible (see details in Supplemental Information 3).

### Procedure

#### Exposure procedure

Prior to the main study procedure, 46 naïve participants were shown a series of six pictures as part of the exposure phase (Fig. 1). The participants were able to go back and forward through the set of pictures as long as they wanted, with no time limit. Each picture represented a macaque’s facial expression associated with its description and its corresponding emotional state (see Fig. 1 for additional details). These pictures were used only for this exposure phase, and were presented to participants during the online questionnaire after the demographic questions and just prior to undertaking the questions related to the recognition of Barbary macaques’ facial expressions. The exposure phase aimed to simulate the condition in which tourists might receive information about facial expressions of Barbary macaques from a notice board in a tourist site where Barbary macaques are present.

#### Main study procedure

The participants started the questionnaire with a brief of the study, and their consent was requested prior the start of the questionnaire. Data were collected for all participants online using Qualtrics (Qualtrics LLC, Provo, UT, USA). Each participant was asked to identify the emotional state of different Barbary macaques based on their facial expression in a series of high resolution 2D pictures. All participants were exposed to the same 16 pictures, but these pictures were automatically randomised by Qualtrics for each participant.

The questionnaire was divided into two parts: demographic questions were followed by questions relating to the recognition of Barbary macaques’ facial expressions. For the exposed participants, the exposure phase occurred between these two parts. The demographic questions included the age, gender, and questions related to their experience with Barbary macaques. Following these demographic questions, 16 pictures were presented, one at a time, with the following question: “Could you specify the emotional state of this monkey?” Participants were asked to select only one answer out of eight different possibilities: very friendly, friendly, neutral, distressed, very distressed, aggressive, very aggressive, other-please specify. When participants selected “other-please specify”, they had to choose an adjective that described best the emotional state of the monkey. If the adjective they chose was closely related to one of the other categories, their response was merged with the responses of this category. For example, some participants referred to distressed pictures as “fear”, thus such answers were re-categorised as distressed. However, when participants chose an adjective that did not obviously relate to any other categories (e.g., “tired”), such answers were counted as “other”. In addition, the intensity of the emotional states was not taken into account in the present analyses; therefore all answers from the categories that included the adverb “very” prior to the emotional state were merged with the corresponding emotional state (e.g., “very friendly” and “friendly” were merged during the data analysis).

### Statistical analysis

We used a generalised linear mixed model (GLMM) to investigate whether participants’ ability to recognise each macaque’s facial expression was predicted by their experience (i.e., between subjects: expert, exposed, naïve) or the type of emotional states (i.e., within subjects: aggressive, distressed, friendly or neutral). The dependent variable, participants’ ability to recognise facial expressions, was binary (i.e., yes or no). The independent variables, i.e., experience and emotion, were categorical. We included as random factors the participants’ and pictures’ identification numbers. The GLMM was run using R 3.1.3 (R Development Core Team, 2015), using the function glmer of the R-package lme4, family = “binomial” for binomial mixed-effects models (Bates & Maechler, 2010). The significance of the individual independent variables was determined based on the *z*- and *p*-values provided by lmer. The significance of the full model was compared to the corresponding null model, containing only the dependent variable and the two random factors, using a likelihood ratio test (R function ANOVA, Bolker et al., 2009). We checked that the model did not violate any assumptions of collinearity, with all variance inflation factors <2. We tested the over dispersion of the data and we accepted the model if the result was equal to 1.

We then calculated the percentage of correct choices for each participant across the four emotions. These data were not normally distributed (i.e., Kolmogorov–Smirnov tests were all *p* < 0.05); therefore, we used non-parametric Mann–Whitney *U* tests. We ran a series of pairwise comparisons between the three categories (naïve, exposed and experts) for the percentage of correct choices and for each type of emotions using SPSS v22 (©IBM).

Finally we described the choice made by the participants for the aggressive and distressed pictures using a confusion matrix. We ran a series of Chi Squared tests and Mann–Whitney *U* tests to assess whether participants confused aggressive and/or distressed pictures with either neutral or friendly faces based on their experience. These analyses were run using SPSS v22 (©IBM).

## Results

**Figure 2:**
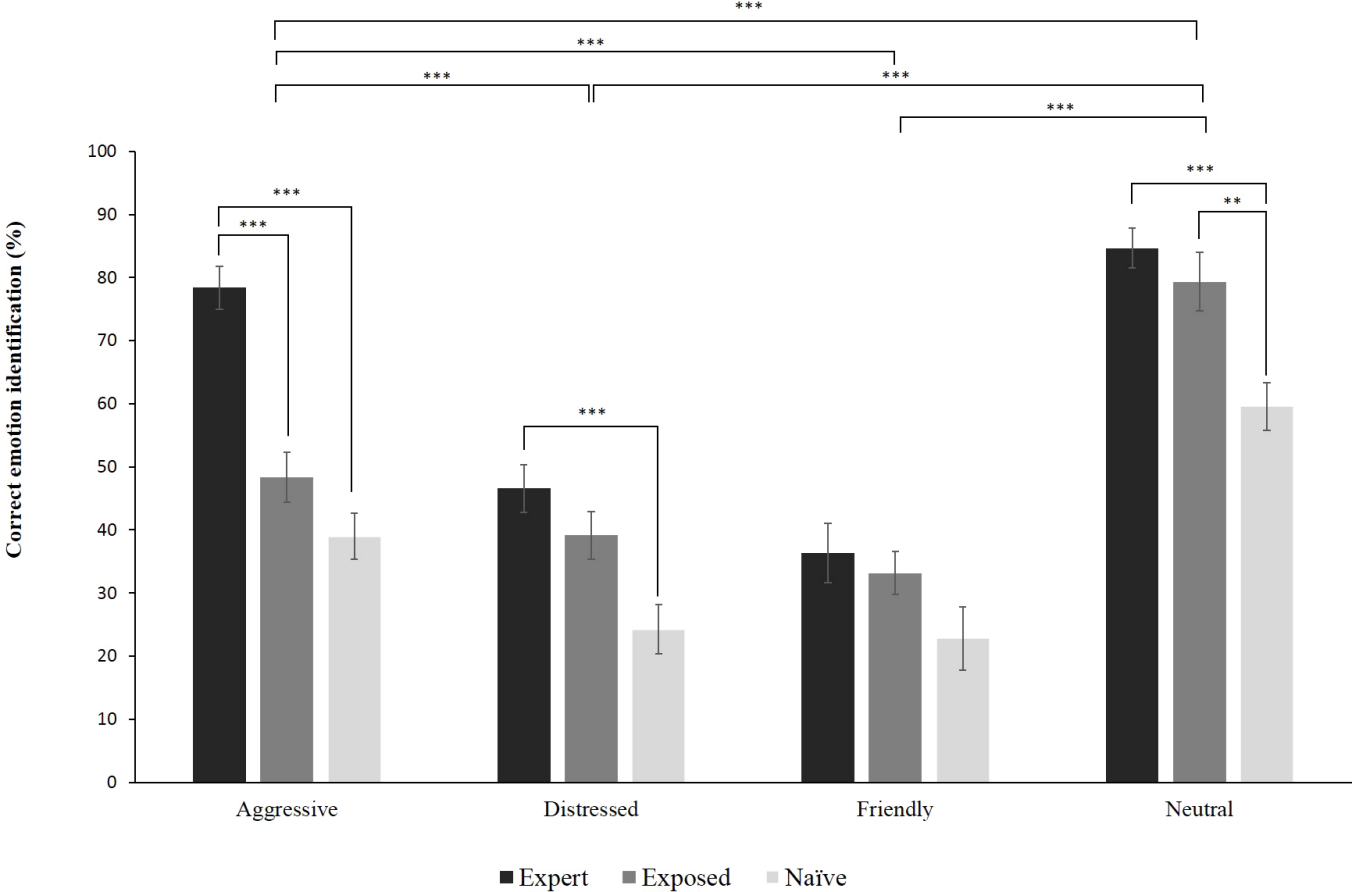
Bar graph representing the percentage of correct identification scores of Barbary macaques’ emotional state by experience and emotional states. *** *p* = 0.01, ** *P* < 0.01, * *P* < 0.05. Error bars represent ±1 standard error of the mean.

Experience significantly predicted participants’ ability to recognise macaques’ emotional states based on their facial expressions, with performance decreasing from expert > exposed > naïve (Table 1, Fig. 2). In addition, there was a significant difference in the participants’ performance dependent upon the type of emotional states with the following descending order: neutral > aggressive > distressed and friendly. Performance on distressed and friendly faces was not significantly different (Table 1).

**Table 1:** Results of the GLMM testing the difference in participants’ abilities to correctly assess macaque’s emotional state based on their experience and the types of emotion. Bold values show statistically significant *P* values (*P* < 0.05).

Full vs. null	*N*	χ2	df	*p*
	1984	204.19	4	**<0.001**
	**Estimate**	**±SE**	***z***	***p***
Intercept	0.832	1.906	4.368	**<0.001**
**Experience**				
Expert vs. Naïve	−1.232	0.157	−7.868	**<0.001**
Expert vs. Exposed	−0.560	0.141	−3.962	**<0.001**
Exposed vs. Naïve	−0.672	0.153	−4.391	**<0.001**
**Emotion**				
Aggressive vs. Distressed	−0.841	0.238	−3.542	**<0.001**
Aggressive vs. Friendly	−1.142	0.239	−4.772	**<0.001**
Aggressive vs. Neutral	0.966	0.243	3.979	**<0.001**
Distressed vs. Friendly	−0.301	0.239	−1.257	0.209
Distressed vs. Neutral	1.807	0.245	7.389	**<0.001**
Friendly vs. Neutral	2.108	0.247	8.548	**<0.001**

The performances were differently affected by the level of experience for each type of emotional states (Table 2, Fig. 2). Experts performed better compared to exposed and naïve participants for the aggressive emotion. Experts also performed better for the distressed and neutral emotional states compared to naïve participants, but not exposed participants. In addition, exposed participants had higher performance for the neutral and distressed facial expressions than naïve participants. However, there were no significant differences in performance between participants for the friendly faces, and between exposed and naïve participants for the aggressive faces (Table 2).

**Table 2:** Results of the Mann–Whitney *U* tests exploring the difference between participants’ expertise to correctly identify the different emotional states. Bold values show statistically significant *P* values (*P* < 0.05).

	*N*	***U***	***Z***	***p***
**Expert vs. Exposed**	**90**			
Aggressive		399.5	−5.132	**<0.001**
Distressed		854.5	−1.329	0.184
Friendly		937.0	−0.626	0.531
Neutral		903.5	−0.963	0.335
**Expert vs. Naïve**	** 78**			
Aggressive		191.5	−5.780	**<0.001**
Distressed		409.0	−3.566	**<0.001**
Friendly		576.0	−1.807	0.071
Neutral		360.5	−4.113	**<0.001**
**Exposed vs. Naïve**	** 80**			
Aggressive		607.5	−1.803	0.071
Distressed		549.5	−2.355	**0.019**
Friendly		687.0	−0.967	0.334
Neutral		454.5	−3.327	**0.001**

We investigated the errors made by each participant group for the aggressive faces (Table 3). We found that aggressive faces were mistaken with both neutral and friendly faces (Expert: 6.8%, Exposed: 20.7%, and Naïve: 37.5%), meaning that exposed and naïve participants are particularly susceptible to mistaking aggressive displays with non-threatening faces. There was a significant difference between levels of experience in mistakenly assessing aggressive faces for friendly and neutral faces (friendly: *N* = 124, χ^2^ = 25.870, *p* < 0.001; neutral: *N* = 124, χ^2^ = 14.681, *p* < 0.001). Experts made fewer mistakes associating aggressive faces with friendly faces than exposed and naïve participants (Expert vs. Exposed: Mann Whitney *U* test: *N* = 90, *U* = 640, *p* < 0.001; Expert vs. Naïve: *N* = 78, *U* = 363, *p* < 0.001). Experts also made fewer errors associating aggressive faces with neutral faces than naïve participants (*N* = 78, *U* = 501.5, *p* = 0.002), but not exposed participants (*N* = 90, *U* = 998, *p* = 0.853). Exposed participants made fewer mistakes associating aggressive faces with neutral faces than naïve participants (*N* = 80, *U* = 531.5, *p* = 0.002), but there was no significant difference with friendly faces (*N* = 80, *U* = 659, *p* = 0.189).

**Table 3:** Confusion matrix of the percentage of correct and incorrect assessment of the different emotional states by level of expertise (excluding “other” category). Highlighted in grey the percentage of correct identification of the emotional states.

Experience	Actual	Predicted			
		Aggressive	Distressed	Friendly	Neutral
Expert	Aggressive	78.41	13.07	5.11	1.70
	Distressed	21.59	46.59	15.91	13.64
	Friendly	5.68	53.41	36.36	3.98
	Neutral	2.27	2.84	7.39	84.66
Exposed	Aggressive	48.37	30.43	15.76	4.89
	Distressed	38.04	39.13	16.85	5.98
	Friendly	27.72	35.33	33.15	3.80
	Neutral	0.54	10.33	9.78	79.35
Naïve	Aggressive	38.97	21.32	15.44	22.06
	Distressed	39.71	24.26	22.06	13.97
	Friendly	28.68	29.41	22.79	19.12
	Neutral	4.41	22.06	12.50	59.56

For distressed faces, participants identified them correctly in less than 50% of the pictures presented (Table 3). We found that all participants were likely to misinterpret distressed faces for non-threatening faces, i.e., friendly and neutral, in 29.5% for experts, in 22.8% for exposed, and 37.5% of naïve participants. There was a significant difference between levels of experience in mistakenly associating distressed faces with neutral faces (*N* = 124, χ^2^ = 9.477, *p* = 0.009), but not with friendly faces (*N* = 124, χ^2^ = 2.375, *p* = 0.305). Naïve participants made more mistakes associating distressed faces with neutral faces than experts and exposed participants (Expert vs. Naïve: Mann Whitney *U* test: *N* = 78, *U* = 508, *p* = 0.003; Exposed vs. Naïve: *N* = 80, *U* = 544.5, *p* = 0.004). There was no significant difference between experts and exposed participants in the number of errors they made associating distressed faces with neutral faces (*N* = 90, *U* = 1002, *p* = 0.927).

## Discussion

The ability to recognize others’ emotional state is an important factor that facilitates social interactions, and facial expressions are one of the main vectors of such information in primates. Interspecies communication, especially when humans interact with animals, is also highly reliant on facial cues to interpret the emotional states of the animals involved. We explored whether and how experience and type of emotions affect the discrimination of emotions in a close phylogenetic species to humans, the Barbary macaque, using photographs of faces. Overall, our results indicated that experience is associated with better rates of recognition of emotional states of Barbary macaques. The results also indicated that exposed and naïve participants had the highest rates of errors associating aggressive faces with neutral or friendly faces. In addition, all participants confused distressed faces with other emotional states (mistake rates > 50%).

Experienced participants who have worked with Barbary macaques appeared to perform better than exposed and naïve participants when assessing the emotional state of macaques based on their facial expressions. Experts were able to recognise aggressive faces better than exposed and naïve participants, and were able to identify distressed and neutral faces better than naïve, but not exposed participants. However, there was no significant difference between participants in their ability to assess friendly faces. These results are in line with previous findings in dogs, where experience seems to be associated with better performance in assessing dog facial expression, although the effect appears to be small (Bloom & Friedman, 2013; Schirmer, Seow & Penney, 2013; Kujala et al., 2017). Experience might also explain the cross-cultural differences that affect performance in human facial recognition of emotions. A number of studies have recently provided evidence that facial expressions of emotions are not universal and that cultural differences exist in the ability to recognise emotional states based on facial expression of other cultural and ethnic groups (Jack et al., 2012; Yan et al., 2016). Barbary macaques have been shown to have a similar facial morphology to other primates, enabling them to produce homologous facial expressions (Julle-Daniere et al., 2015). However, the use of facial expressions seems to be specific to the communicative repertoire of the species (Preuschoft & Van Hooff, 1997; Julle-Daniere et al., 2015).

Participants’ performance differed significantly between emotions, highlighting the difficulty in discriminating distressed and friendly faces in Barbary macaques across all experience levels, and aggressive faces in exposed and naïve participants. These findings differ from previous research in humans and dogs, where happiness was one of the emotions that was easily recognised compared to neutral or fear faces (e.g., humans: Durand et al., 2007; dogs: Bloom & Friedman, 2013). In addition, previous studies of facial expressions in humans have also shown that angry human faces were recognised more easily than other expressions, such as neutral facial displays (Hansen & Hansen, 1988; Fox et al., 2000; Calvo, Avero & Lundqvist, 2006). It is thought that angry faces are used to signal the presence of a potential threat (e.g., aggression received); thus, the ability to quickly detect such faces would enhance better survival. Experience seems to improve the recognition of aggressive and distressed faces, but experience does not appear to be sufficient to recognise distressed and friendly faces above chance levels. These emotional states might be expressed in a sequence of behaviours or facial expressions, and so the ability of recognising these emotions might increase by using video rather than static pictures. For example teeth chattering in Barbary macaque is a quick succession of movement where the upper and lower teeth hit against each other, often associated with a clicking noise. Taking a picture of such behaviour may therefore not give enough dynamic clues to efficiently recognise the facial expression and thus, interpret the emotional state of the animal.

Overall, our results indicate that experience and type of emotions are key factors in the ability to recognise emotions in Barbary macaque facial expressions. These findings do not support the universality hypothesis (Ekman, 1992; Ekman, 1993), which suggest the evolutionary origins of the six basic emotions. Therefore, the specificity of the communicative repertoire between species or cultural groups might highly rely on experience and type of emotions rather than universal principles.

### Applied perspective: implications for primate tourism

Even though it is commonly prohibited, interacting closely with wild animals, such as feeding and physical contact, is a very popular activity in wildlife tourism, and can also occur in zoos or animal parks despite the enclosure. However, such interactions present a potential risk of injury for both tourists and the animals involved (Zhao & Deng, 1992; Paterson & Wallis, 2005; Maréchal et al., 2016b). Therefore, the ability to recognise the emotional state of an animal people interact with, can reduce such risk. This is particularly important for aggressive or distressed faces, which, if ignored, are more likely to trigger inappropriate interactions and hence potential injuries.

Short exposure to pictures of Barbary macaques’ faces increases the ability of the participants to recognise monkey emotion. However, the performance rates of exposed participants were under 50% chance levels when assessing aggressive, distressed and friendly faces. There was also a significant difference between experts and exposed participants in discriminating aggressive faces. Taken together, these findings indicate that simply exposing people to Barbary macaque facial expressions and their corresponding emotional states using pictures on a board as is routinely done in a wildlife tourism or zoo setting, might not be a sufficient educational tool to reduce misinterpretation of an animal’s emotional state. The presentation of different facial orientation might make the recognition more difficult. Therefore, people should be exposed to similar stimuli taken from different angles to evaluate whether increased experience on different tilted faces improve their recognition abilities. As previously stated, learning might also be improved by showing a sequence of behaviours either live or on video, as the movement or context in which facial expressions are displayed can give additional clues that can help people to discriminate macaques’ emotional states.

Participants confused aggressive faces with ‘distressed,’ neutral and friendly faces; this was particularly true for exposed and naïve participants. Experts made 20.2% mistakes in interpreting aggressive facial expressions, whereas exposed and naïve people were even more likely to misinterpret aggressive faces (exposed: 51.4%, naïve: 60.2%) which might lead to situations where inappropriate behaviour towards the macaques is shown, increasing the risk of injury. Anecdotally, one of us (LM) often heard tourists in Morocco saying that the monkey seemed to blow them a kiss when they actually displayed a threatening face (Fig. 1B). The tourists often responded by imitating the monkey’s facial expression, which generally ended by either an aggression given by the monkey towards the tourists or the monkey leaving the interaction. Such a misunderstanding of Barbary macaque emotion can be related to an interpretative anthropomorphism, when people attribute intentions and emotions to nonhuman animals based on their behaviour (Fisher, 1991). This is particularly common for species that are phylogenetically close to humans, such as nonhuman primates (Urquiza-Haas & Kotrschal, 2015).

All participants confused distressed faces with other emotional states. In all the distressed faces presented, the teeth were visible with the lips fully retracted, with or without an open mouth. Showing teeth with the lips fully retracted is often considered as threatening in a number of animal species such as dogs (Tami & Gallagher, 2009), rodents (Zenuto, Vassallo & Busch, 2001), or as angry in humans (Pinkham et al., 2010). Therefore, this might explain why the participants confused distressed faces for aggressive faces. In addition, all participants have also mistaken distressed faces with neutral or friendly faces. We included in the questionnaire a picture of a yawning macaque, which we classified as distressed face. Variations in rates of yawning as well as other self-directed behaviours, such as self-scratching, have been found to reflect animal emotional state such as anxiety or frustration, generally displayed during stress-related situations (Maestripieri et al., 1992). Pharmacological and behavioural studies lend strong support to this idea (Schino et al., 1996; Castles, Whiten & Aureli, 1999; Troisi, 2002). For instance, increasing rates of self-scratching were positively related to interacting with tourists in wild male Barbary macaques, suggesting that male macaques had higher levels of anxiety when interacting with tourists (Maréchal et al., 2011; Maréchal et al., 2016a). However, the participants might not have been aware of the association between yawning and self-directed behaviours, and distressed emotional state in animals, which would explain this result. They might also have mistaken the showing teeth for a “smile” as displayed in humans (Preuschoft, 1992; Preuschoft & Van Hooff, 1997). This mistake is also observed in children who mistake a dog’s display of teeth as a happy and smiling facial expression (Meints, Racca & Hickey, 2011). However, being able to recognise such distressed faces or behaviour in animals, as well as knowing in which contexts tourists might elicit such facial expressions, are important in order to propose appropriate measures that reduce the costs for animal welfare, while improving tourist experience. In humans, when confronted with fearful faces, participants were more likely to approach than avoid the stimuli (Marsh, Ambady & Kleck, 2005; Adams et al., 2006). Only one study has so far been conducted using dog faces as stimuli (Meints, Racca & Hickey, 2011), but none using nonhuman primates. However, there is anecdotal evidence that shows tourists sometimes initiate an interaction with wild animals because they perceive them in distress, and this may lead to a worse situation for the animals. Future research should explore human responses to animals perceived as distressed or fearful, and educational measures should be initiated at tourist sites to ensure that people do not approach such animals in order to reduce the risk of injury.

Overall, the present study found that experience helps in correctly decoding Barbary macaque facial signalling, but a simple exposure to such stimuli using pictures is not efficient enough to significantly improve the performance of exposed and naïve people above chance (50%) for aggressive, distressed and friendly faces. This is an important issue, because these people are likely to be the tourists who encounter or interact with Barbary macaques in different wildlife tourism settings. Therefore, we propose some measures that aim to mitigate the potential risks for the tourists who have low understanding of animals’ behaviour and/or facial expressions. Actual training of tourists or guided visits by experienced professionals may be required to acquire a recognition rate that would effectively reduce misinterpretation of primate emotion based on facial expression, and thus decrease the risk of injury. These guided visits by local professionals can also benefit the local economy and increase public awareness, leading to a better conservation of the species involved. A successful example of the positive impact of guided visits by local experts can be found in gorilla tourism (Williamson & Macfie, 2010; Nielsen & Spenceley, 2012).

It would also be interesting to test in future research whether a more interactive training such as video or participative games increase the ability of inexperienced people to assess Barbary macaque emotion. Extensive information of an animal’s emotional state can also be conveyed by posture and vocalisations (Seyfarth & Cheney, 2003; Tami & Gallagher, 2009); therefore, including other sources of information in addition to facial clues might increase the performance of inexperienced people. Interactive training would be useful particularly for zoo settings. However, such a recommendation is unlikely to be feasible in a number of wildlife tourism settings; therefore, we suggest in addition to guided visits, regulating the distance between the tourists and wild animals as keeping a safe distance will reduce such risks. In addition to increased safety for tourists, these recommendations can improve animal welfare. Indeed, it has previously been found in a number of species that the presence, proximity and/or interactions with tourists can negatively affect animal welfare by increasing their physiological stress levels (e.g., Magellanic penguin, *Spheniscus magellanicus*: Fowler, 1999; Barbary macaque: Maréchal et al., 2011; western lowland gorilla, *Gorilla gorilla gorilla*: Shutt et al., 2014), risks of injuries related to higher aggression and conflicts with conspecifics (e.g., stingray, *Dasyatis americana*: Semeniuk & Rothley, 2008; Barbary macaques: Majolo et al., 2013) and tourists (e.g., Tibetan macaques, *Macaca Thibetana*: Zhao & Deng, 1992; stingray: Lewis & Newsome, 2003). Therefore, to reduce human-animal conflicts, preventing inappropriate behaviour from tourists towards animals is crucial and this improves animal welfare and tourist experience simultaneously. Understanding how people perceive animal emotion and behaviour, and how they respond to them will be key in future wildlife and zoo tourism research.

##  Supplemental Information

10.7717/peerj.3413/supp-1 Data S1 Dataset

10.7717/peerj.3413/supp-2 Supplemental Information 2 Questionnaire

10.7717/peerj.3413/supp-3 Supplemental Information 3 Pilot study

## Additional Information and Declarations

### Data Availability

The following information was supplied regarding data availability: The raw data has been supplied as a Supplementary File.
