# Supplementary material for: Experience-based human perception of facial expressions in Barbary macaques (Macaca sylvanus)
Source: PeerJ. 2017 Jun 1;5:e3413. doi: 10.7717/peerj.3413 (PMC5457665; doi:10.7717/peerj.3413)
Supplement: Supplemental Information 2 [file peerj-05-3413-s002.pdf]

Questionnaire on human perception of Barbary macaques’ facial expressions

Brief
This study aims to analyse how humans rate different facial expressions of Barbary macaques. A questionnaire will be displayed on your computer screen, showing pictures of Barbary macaques' faces. Please choose in both questions which emotional state better describes each monkey's face.

Please confirm: You agree to take part in this research. You are aware that you are free to withdraw at any point. You understand that any information you provide will be treated in confidence by the investigators. You will stay anonymous in the publication of any findings.

<u>Questionnaire part 1</u>

Q1 I consent to participate in this study
❍ Yes (1)
❍ No (2)

Q2 Age
❍ 18-25 (1)
❍ 26-40 (2)
❍ 41-60 (3)
❍ 61+ (4)

Q3 Gender
❍ Male (1)
❍ Female (2)
❍ Other (3)

Q4 Have you ever seen live non-human primates in real life before?
❍ Yes (1)
❍ No (2)

Display This Question:
If Have you had any previous experience with primates? Yes Is Selected

Q4 Which species? (Please give the names of 1or 2 species you have seen the most / or you have worked with)

Display This Question:
If Have you ever encountered live primates before? Yes Is Selected

Q5 In which context?
❍ Work (1)
❍ Holiday (2)
❍ All of the above (4)
❍ Other, please specify (3) ____________________

Display This Question:
If In which context? Work Is Selected
Or In which context? All of the above Is Selected

Q6 How long have you worked with non-human primates?

Display This Question:
If In which context? Work Is Selected
Or In which context? All of the above Is Selected

Q7 Which type of work have you conducted?
- ❍ Behavior (1)
- ❍ Other, please specify (2) ____________________

Q8 Have you ever seen live Barbary macaques in real life before?
- ❍ Yes (1)
- ❍ No (2)

Display This Question:
If Have you had any experience with Barbary Macaques? Yes Is Selected

Q9 In which context have you seen live Barbary macaques?
- ❍ Work (1)
- ❍ Holiday (2)
- ❍ All of the above (4)
- ❍ Other, please specify (3) ____________________

Display This Question:
If In which context? Work Is Selected
Or In which context? All of the above Is Selected

Q10 How long have you worked with Barbary macaques?

Display This Question:
If In which context? Work Is Selected
Or In which context? All of the above Is Selected

Q11 Which type of work have you conducted?
- ❍ Behavioural (1)
- ❍ Other, please specify (2) ____________________

Display This Question:
If Have you had any experience with Barbary Macaques? No Is Selected

Q12 Have you ever seen a Barbary macaque before, in a picture?
- ❍ Yes (1)
- ❍ No (2)

Display This Question:
If Have you had any experience with Barbary Macaques? No Is Selected

Q13 Have you ever seen a Barbary macaque before, in a video/film?
- ❍ Yes (1)
- ❍ No (2)

Display This Question:

If Have you ever seen a Barbary macaque before, in a picture? Yes Is Selected

Or Have you ever seen a Barbary macaque before, in a video/film? Yes Is Selected

Q14 In which context?

- Work (1)
- Holiday (2)
- Other, please specify (3) ____________________

Display This Question:

If In which context Work Is Selected

Q15 Which type of work have you conducted?

- Behavioural (1)
- Other, please specify (2) ____________________

Please note: if you have any concerns about any aspect of your participation or any other queries please raise this with the investigators. Contact details: Dr Laetitia Marechal or Xandria Levy School of Psychology, College of Social Science, University of Lincoln, Brayford Pool, Lincoln, Lincolnshire LN6 7TS Tel: +44 (0)1522 837409
Email: facialexpressionsofbarbarymacaques@outlook.com

Training phase

This is the training phase. Please find below examples of Barbary macaques' faces, with a label naming the type of facial expression displayed over each picture. Some definitions are extracted from Teufel et al. (2010).

Neutral Facial Expression: the mouth is closed and the overall face is relaxed.

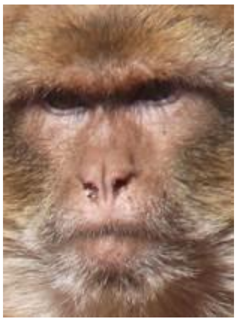

Friendly or 'Affiliative' Facial Expression: the mouth is half open and the lips slightly protruded. This expression involves a chewing movement and clicking or smacking of the tongue and lips.

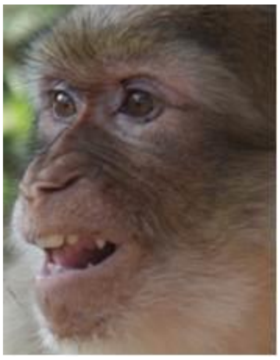

Distressed Facial Expression: the mouth is widely open, and the animal is yawning. Yawing might be expressed when an animal is in a discomforting situation.

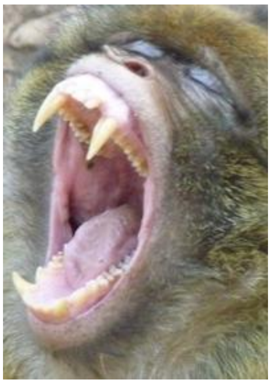

Very Distressed or 'Submissive' Facial Expression: the corners of the lips are fully retracted and the upper and lower teeth are shown.

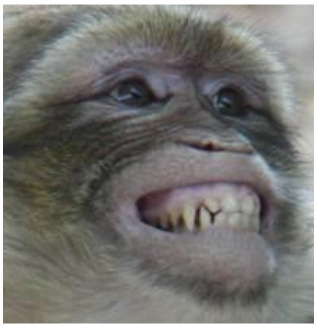

Aggressive or 'Threat' Face: the eyebrows are raised, the animal stares intently and the lips are protruded to form a round mouth.

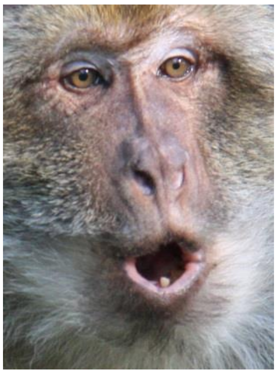

Very Aggressive Facial Expression: the eyebrows are raised, the animal stares intently and the mouth is open showing the teeth.

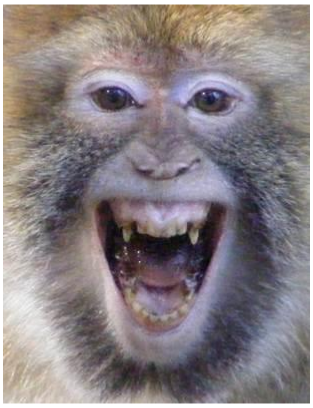

<u>Questionnaire part 2</u>

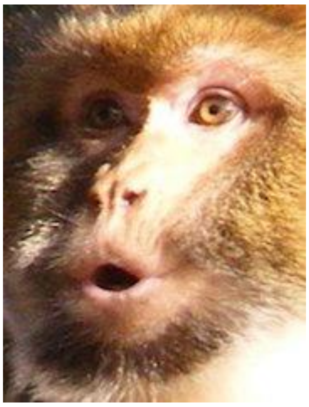

Q16 Could you specify the emotional state of this monkey?

- ❍ Very friendly (1)
- ❍ Friendly (2)
- ❍ Neutral (3)
- ❍ Distressed (4)
- ❍ Very Distressed (5)
- ❍ Aggressive (6)
- ❍ Very Aggressive (7)
- ❍ Other, please specify (8) ____________________

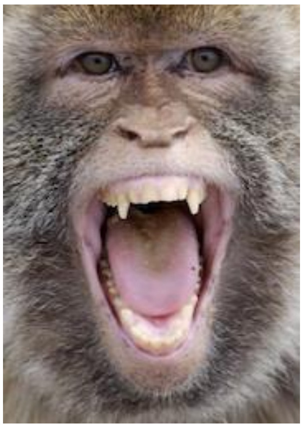

Q17 Could you specify the emotional state of this monkey?

- ❍ Very friendly (1)
- ❍ Friendly (2)
- ❍ Neutral (3)
- ❍ Distressed (4)
- ❍ Very Distressed (5)
- ❍ Aggressive (6)
- ❍ Very Aggressive (7)
- ❍ Other, please specify (8) ____________________

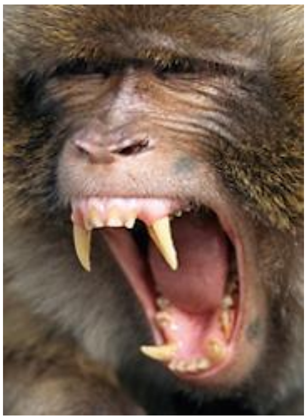

Q18 Could you specify the emotional state of this monkey?

- ❍ Very friendly (1)
- ❍ Friendly (2)
- ❍ Neutral (3)
- ❍ Distressed (4)
- ❍ Very Distressed (5)
- ❍ Aggressive (6)
- ❍ Very Aggressive (7)
- ❍ Other, please specify (8) ____________________

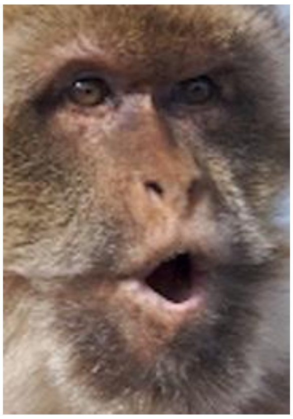

Q19 Could you specify the emotional state of this monkey?

- ❍ Very friendly (1)
- ❍ Friendly (2)
- ❍ Neutral (3)
- ❍ Distressed (4)
- ❍ Very Distressed (5)
- ❍ Aggressive (6)
- ❍ Very Aggressive (7)
- ❍ Other, please specify (8) ____________________

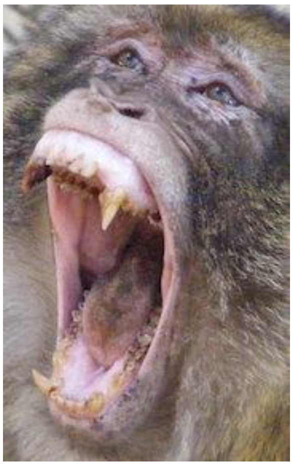

Q20 Could you specify the emotional state of this monkey?

- ❍ Very friendly (1)
- ❍ Friendly (2)
- ❍ Neutral (3)
- ❍ Distressed (4)
- ❍ Very Distressed (5)
- ❍ Aggressive (6)
- ❍ Very Aggressive (7)
- ❍ Other, please specify (8) ____________________

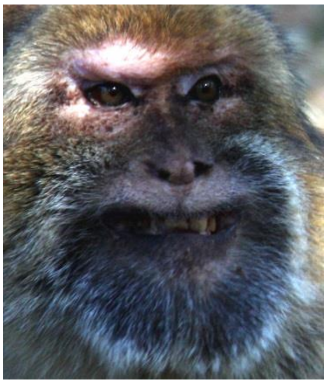

Q21 Could you specify the emotional state of this monkey?

- ❍ Very friendly (1)
- ❍ Friendly (2)
- ❍ Neutral (3)
- ❍ Distressed (4)
- ❍ Very Distressed (5)
- ❍ Aggressive (6)
- ❍ Very Aggressive (7)
- ❍ Other, please specify (8) ____________________

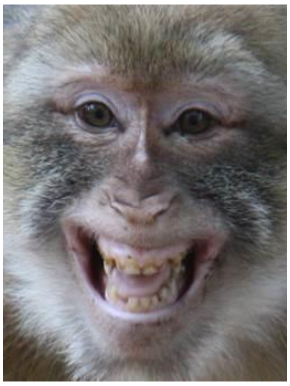

Q22 Could you specify the emotional state of this monkey?

- Very friendly (1)
- Friendly (2)
- Neutral (3)
- Distressed (4)
- Very Distressed (5)
- Aggressive (6)
- Very Aggressive (7)
- Other, please specify (8) ____________________

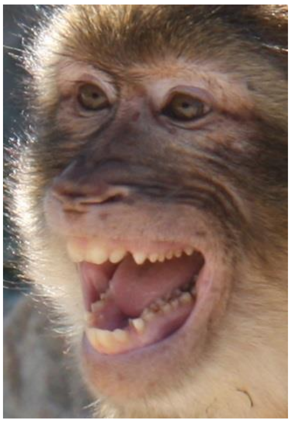

Q23 Could you specify the emotional state of this monkey?

- Very friendly (1)
- Friendly (2)
- Neutral (3)
- Distressed (4)
- Very Distressed (5)
- Aggressive (6)
- Very Aggressive (7)
- Other, please specify (8) ____________________

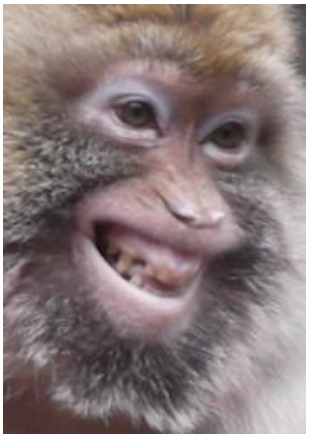

Q24 Could you specify the emotional state of this monkey?

- ❍ Very friendly (1)
- ❍ Friendly (2)
- ❍ Neutral (3)
- ❍ Distressed (4)
- ❍ Very Distressed (5)
- ❍ Aggressive (6)
- ❍ Very Aggressive (7)
- ❍ Other, please specify (8) ____________________

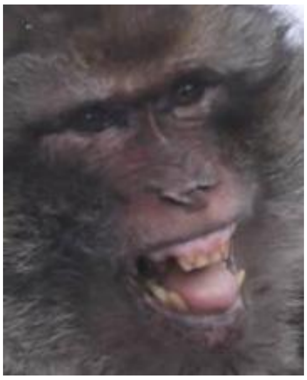

Q25 Could you specify the emotional state of this monkey?

- ❍ Very friendly (1)
- ❍ Friendly (2)
- ❍ Neutral (3)
- ❍ Distressed (4)
- ❍ Very Distressed (5)
- ❍ Aggressive (6)
- ❍ Very Aggressive (7)
- ❍ Other, please specify (8) ____________________

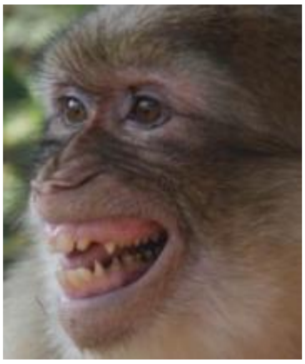

Q26 Could you specify the emotional state of this monkey?

- Very friendly (1)
- Friendly (2)
- Neutral (3)
- Distressed (4)
- Very Distressed (5)
- Aggressive (6)
- Very Aggressive (7)
- Other, please specify (8) ____________________

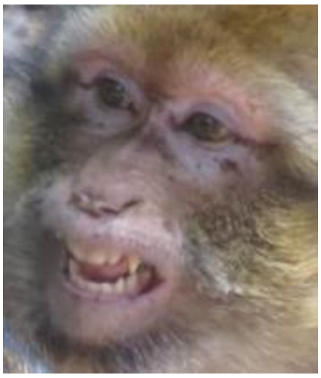

Q27 Could you specify the emotional state of this monkey?

- Very friendly (1)
- Friendly (2)
- Neutral (3)
- Distressed (4)
- Very Distressed (5)
- Aggressive (6)
- Very Aggressive (7)
- Other, please specify (8) ____________________

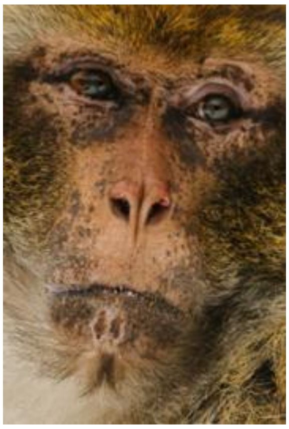

Q28 Could you specify the emotional state of this monkey?

- ❍ Very friendly (1)
- ❍ Friendly (2)
- ❍ Neutral (3)
- ❍ Distressed (4)
- ❍ Very Distressed (5)
- ❍ Aggressive (6)
- ❍ Very Aggressive (7)
- ❍ Other, please specify (8) ____________________

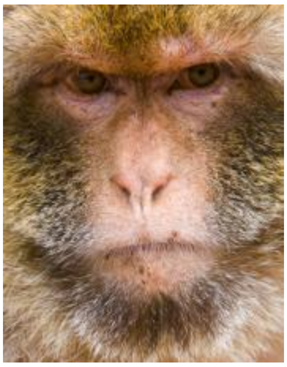

Q29 Could you specify the emotional state of this monkey?

- ❍ Very friendly (1)
- ❍ Friendly (2)
- ❍ Neutral (3)
- ❍ Distressed (4)
- ❍ Very Distressed (5)
- ❍ Aggressive (6)
- ❍ Very Aggressive (7)
- ❍ Other, please specify (8) ____________________

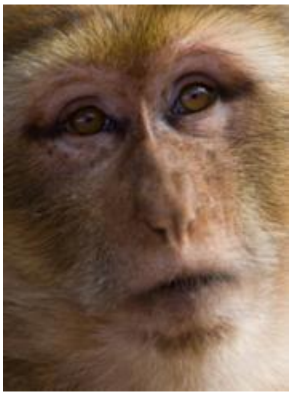

Q30 Could you specify the emotional state of this monkey?
- ❍ Very friendly (1)
- ❍ Friendly (2)
- ❍ Neutral (3)
- ❍ Distressed (4)
- ❍ Very Distressed (5)
- ❍ Aggressive (6)
- ❍ Very Aggressive (7)
- ❍ Other, please specify (8) ____________________

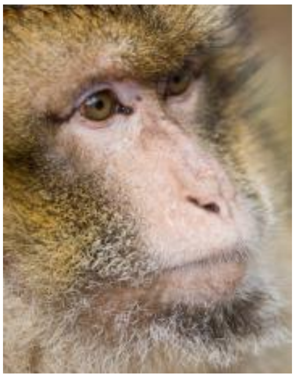

Q31 Could you specify the emotional state of this monkey?
- ❍ Very friendly (1)
- ❍ Friendly (2)
- ❍ Neutral (3)
- ❍ Distressed (4)
- ❍ Very Distressed (5)
- ❍ Aggressive (6)
- ❍ Very Aggressive (7)
- ❍ Other, please specify (8) ____________________

Debrief

Thank you very much for taking part in this study, if you have any questions or comments please contact: facialexpressionsofbarbarymacaques@outlook.com

We would like to sincerely thank Prof. Julia Fischer, Roger Eritja, and Andrew Forsyth for the permission to use their photographs.
