# Supplementary material for: Experience-based human perception of facial expressions in Barbary macaques (Macaca sylvanus)
Source: PeerJ. 2017 Jun 1;5:e3413. doi: 10.7717/peerj.3413 (PMC5457665; doi:10.7717/peerj.3413)
Supplement: Supplemental Information 3 [file peerj-05-3413-s003.pdf]

Supplementary material S2_Pilot study

As we aimed to have realistic photographs of Barbary macaques taken in daylight and with a natural background potentially visible, we also carried out a pilot study in order to test participants' ability of recognising Barbary macaques' emotion between colour versus black and white pictures, and with or without a natural background visible. For this, we asked ten naïve participants divided into two groups to assess five pictures. These participants only took part in this pilot study and were not included in the main study. We presented the first group with two pictures in colour, two in black and white, and one without a natural background. We presented the second group with the same pictures, but with the reverse condition, colour pictures became black and white, and *vice versa*, and the picture without background was presented with a background. We then compared the results of both groups using Mann-Whitney tests. The findings showed no significant differences in participants ability to recognise macaques' emotions between colour and black and white pictures (Mann-Whitney: N=10, U= 10.5, P= 0.650), and with or without visible background (Mann-Whitney: N=10, U= 11, P= 0.746). Manipulation of the pictures did not affect the abilities of participants to recognise macaques' emotional states, therefore to preserve as much a naturalistic image as possible, we conducted our study using colour pictures, and some pictures presented a natural background slightly visible.
